# Association between ultrasound-based biliary and parenchymal intrahepatic mass-forming cholangiocarcinoma subtypes and clinicopathological features and survival

**DOI:** 10.1186/s13244-025-02019-0

**Published:** 2025-06-19

**Authors:** Cong-Jian Wen, Hui Liu, Li-Ping Sun, Chong-Ke Zhao, Hao-Hao Yin, Li-Fan Wang, Ming-Rui Zhu, Yi-Kang Sun, Ya-Qin Zhang, Zi-Tong Chen, Xi Wang, Han-Sheng Xia, Hong Han, Hui-Xiong Xu, Bo-Yang Zhou

**Affiliations:** 1 Department of Ultrasound, Zhongshan Hospital, Institute of Ultrasound in Medicine and Engineering, Fudan University, Shanghai, China; 2 Department of Medical Ultrasound, Center of Minimally Invasive Treatment for Tumor, Shanghai Tenth People’s Hospital, Ultrasound Education and Research Institute, School of Medicine, Tongji University, Shanghai, China; 3 Shanghai Engineering Research Center of Ultrasound in Diagnosis and Treatment, Shanghai, China

**Keywords:** Ultrasonography, Cholangiocarcinoma, Bile ducts, Subtypes, Prognosis

## Abstract

**Objective:**

Mass-forming intrahepatic cholangiocarcinomas (MF-ICCs) can be classified into ductal and parenchymal types using magnetic resonance imaging (MRI). We aimed to subclassify MF-ICC into biliary and parenchymal types based on ultrasound (US) findings and to investigate the differences in their contrast-enhanced ultrasound (CEUS) patterns, clinicopathologic features, and prognosis.

**Methods:**

In this study, 141 patients who underwent US with pathologically proven MF-ICC from two hospitals were retrospectively enrolled. MF-ICCs were divided into biliary (bMF-ICCs) and parenchymal MF-ICC (pMF-ICCs) based on the signs of bile duct dilation in US images. Clinicopathological, imaging, and short-term survival data were collected from medical records and compared.

**Results:**

Among 141 patients (61.96 ± 10.15 years, 83 men), bMF-ICCs (33/141, 23.4%) showed significantly more CEA ≥ 5 µg/L (42.4% vs 20.2%, *p* = 0.01), microvascular invasion (54.5% vs 10.2%, *p* < 0.001), lymph node metastasis (48.5% vs 5.6%, *p* < 0.001), bile duct invasion (48.5% vs 5.6%, *p* < 0.001), and high Ki-67 expression (63.6% vs 38.9%, *p* = 0.01) than pMF-ICCs. Pathologically, bMF-ICCs were more inclined toward the large duct type (78.1% vs 11.7%, *p* < 0.001). In addition, the bMF-ICCs were usually located in the left lobe of the liver (63.6% vs 41.7%, *p* = 0.03). pMF-ICCs showed better overall survival than bMF-ICCs (*p* = 0.04).

**Conclusions:**

Subclassification of MF-ICCs into biliary and parenchymal types based on US is useful for discriminating clinicopathological characteristics.

**Critical relevance statement:**

The subclassification of mass-forming intrahepatic cholangiocarcinoma (MF-ICC) into biliary (bMF-ICC) and parenchymal (pMF-ICC) subtypes using ultrasound can provide clinicopathological and prognostic information before surgery.

**Key Points:**

- We subclassified mass-forming intrahepatic cholangiocarcinomas into biliary and parenchymal types using ultrasound.
- Biliary and parenchymal types have different clinicopathological features and postsurgical outcomes.
- Biliary type above and below 50 mm exhibits different unfavorable clinicopathological characteristics.
- Our classification has certain similarities with MRI classification in clinicopathological characteristics.

**Graphical Abstract:**

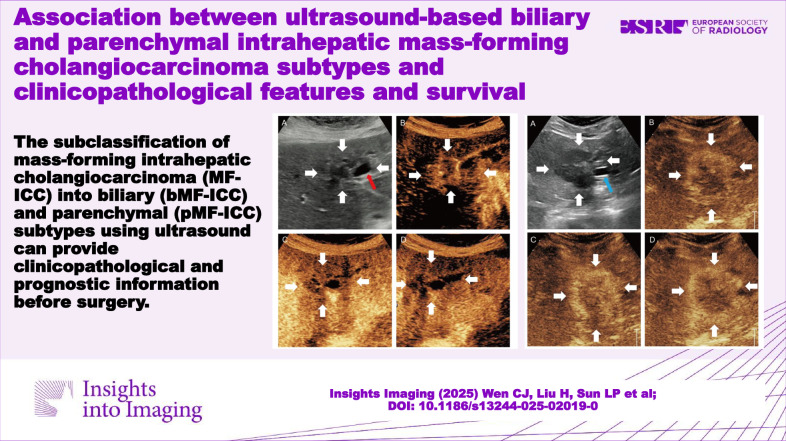

## Introduction

Intrahepatic cholangiocarcinoma (ICC) is the second most common primary malignant tumor of the liver and typically occurs near the secondary bile ducts [1]. It accounts for 20–25% of all bile duct tumors and 20% of all liver malignancies. The incidence of ICC is increasing globally [2–4]. The heterogeneity of ICC tumor cells results in a more complex biological behavior compared to perihilar and distal cholangiocarcinomas [5, 6].

*EASL-ILCA Clinical Practice Guidelines on the Management of Intrahepatic Cholangiocarcinoma (2023 Edition)* classified ICC into four types based on the general morphology for tailored management: mass-forming (MF), periductal-infiltrating (PI), mass formation + periductal infiltration type (MF + PI), and intraductal growth type [7]. According to the *WHO Classification of Digestive System Tumors (5th Edition)*, ICC is further divided into large and small duct types that exhibit distinct clinicopathological features and mutation profiles. In general, the prognosis of small duct-type ICC is significantly better than that of large duct-type ICC, partly because of favorable prospects for targeted therapy, as genetic alterations in IDH1/2 and FGFR2 are predominantly found in small duct-type ICC [8, 9]. In terms of gross classification, small duct-type ICC is typically associated with the MF subtype, whereas large duct types are more often associated with the PI or PI + MF subtypes [8]. The MF type accounts for approximately 60% of all the ICC subtypes, making it the most common morphological classification [10, 11]. However, the MF-ICC can be either small or large duct types, leading to significant variations in prognosis. Therefore, to provide better clinical management for patients with MF-ICC, Rhee et al used MRI to subclassify MF-ICC based on bile duct-related abnormalities [12]. However, MRI is less commonly employed as the initial imaging modality, while visualization of the bile ducts requires multiple sequential scans [13].

US, a radiation-free imaging technique, is a cost-effective method for evaluating abnormalities in the biliary system [14]. However, the prognostic significance of US-based MF-ICC typing remains unclear and requires further investigation. Therefore, this study aimed to subclassify MF-ICCs into biliary (bMF-ICC) and parenchymal (pMF-ICC) MF-ICC based on US findings and to investigate the differences in their contrast-enhanced ultrasound (CEUS) patterns, clinicopathologic features, and postoperative outcomes.

## Methods

### Patients

The institutional Clinical Research Ethics Committee of the participating center (approval numbers: B2022-568R) approved this retrospective study and waived the requirement of informed consent. We retrospectively included consecutive patients with MF-ICC who underwent surgical resection at two hospitals. Patients diagnosed with MF-ICC were enrolled from Zhongshan Hospital, Fudan University (Center 1) between February 2022 and May 2023, and Shanghai Tenth People’s Hospital of Tongji University (Center 2) between January 2020 and March 2023. The tumor location was determined using pathological specimens, with all lesions situated near the secondary bile duct within the liver parenchyma.

The inclusion criteria were as follows: (1) patients diagnosed with focal liver lesions, (2) patients scheduled for surgical resection with a pathological diagnosis of MF-ICC, and (3) patients who underwent conventional US and CEUS examinations within 1 month before surgery and prior to any treatments, including biopsy, radiotherapy, chemotherapy, or other transformative therapies. The exclusion criteria were as follow: (1) lack of clinicopathological information (*n* = 22 at Center 1, *n* = 1 at Center 2), (2) incomplete imaging data (*n* = 11 at Center 1, *n* = 1 at Center 2), (3) significant respiratory motion artifacts resulting in poor US image quality (*n* = 1 at Center 1).

**Fig. 1:**
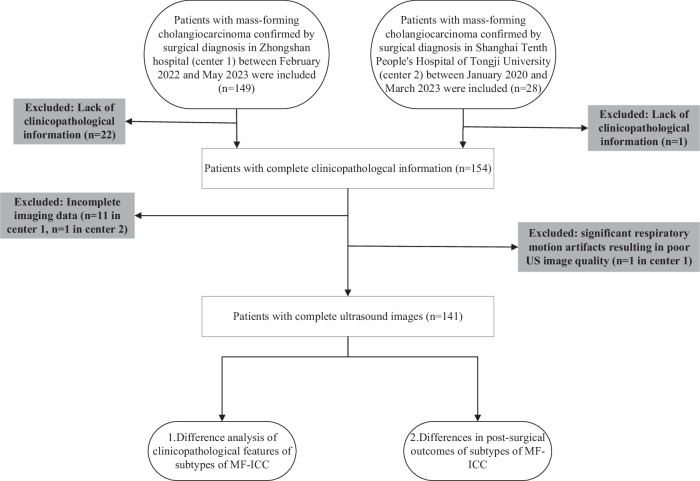
Flowchart of study participants selection in this study

Finally, 141 consecutive patients with MF-ICC were enrolled in this study. A detailed flowchart of patient selection process is shown in Fig. 1*.*

### US image

US examinations were performed by three experienced sonographers specializing in liver US at Center 1 using three types of US instruments (Philips EPIQ7 with a 5C1 convex array transducer, Hitachi Arietta 70 with an EUP-C532 convex array transducer, and Samsung RS80A with a CA1-7A convex array transducer). Three experienced sonographers used GE E9 US instruments with a C1-5 convex array transducer at Center 2. During real-time scanning, the acoustic window and depth were adjusted to ensure simultaneous display. The dynamic range, mechanical index (MI), output power, and focal zone were optimized to achieve effective tissue cancellation while maintaining adequate penetration. Additionally, the MI was set to 0.06–0.08 for CEUS. In this study, conventional US was performed 5–10 min before CEUS. Each patient’s conventional US and CEUS were performed by the same sonographer using the same US machine. A volume of 2.0 (at Center 1) or 1.5 mL (at Center 2) sulfur hexafluoride microbubbles (SonoVue^®^, Bracco Imaging, Milan, Italy) was injected within 1 s via the antecubital vein. A timer was started immediately after the microbubble injection. The enhancement characteristics of MF-ICC during the arterial, portal venous, and late phases were recorded according to the liver CEUS guidelines [15].

The conventional US characteristics recorded included: (1) tumor location (left or right lobe), (2) shape (regular or irregular), (3) echogenicity (hyper-, iso-, or hypo-echogenicity), and (4) margins (clear or ill-defined).

The CEUS characteristics that were recorded included: (1) initial enhancement time (time from contrast agent injection to its appearing in the lesion), (2) time to peak (time from contrast agent injection to the highest enhancement intensity of the lesion), (3) washout time (transition from hyper- or iso-enhancement during the arterial phase to hypo-enhancement), (4) arterial phase enhancement degree (hyper-, iso-, or hypo-enhancement), (5) portal venous phase enhancement degree (hyper-, iso-, or hypo-enhancement), (6) late-phase enhancement degree (hyper-, iso-, or hypo-enhancement), (7) contrast enhancement pattern (circular, non-circular, or nodular enhancement), and (8) degree of clearance (no clearance, slight clearance or significant clearance).

### Images analysis

**Fig. 2:**
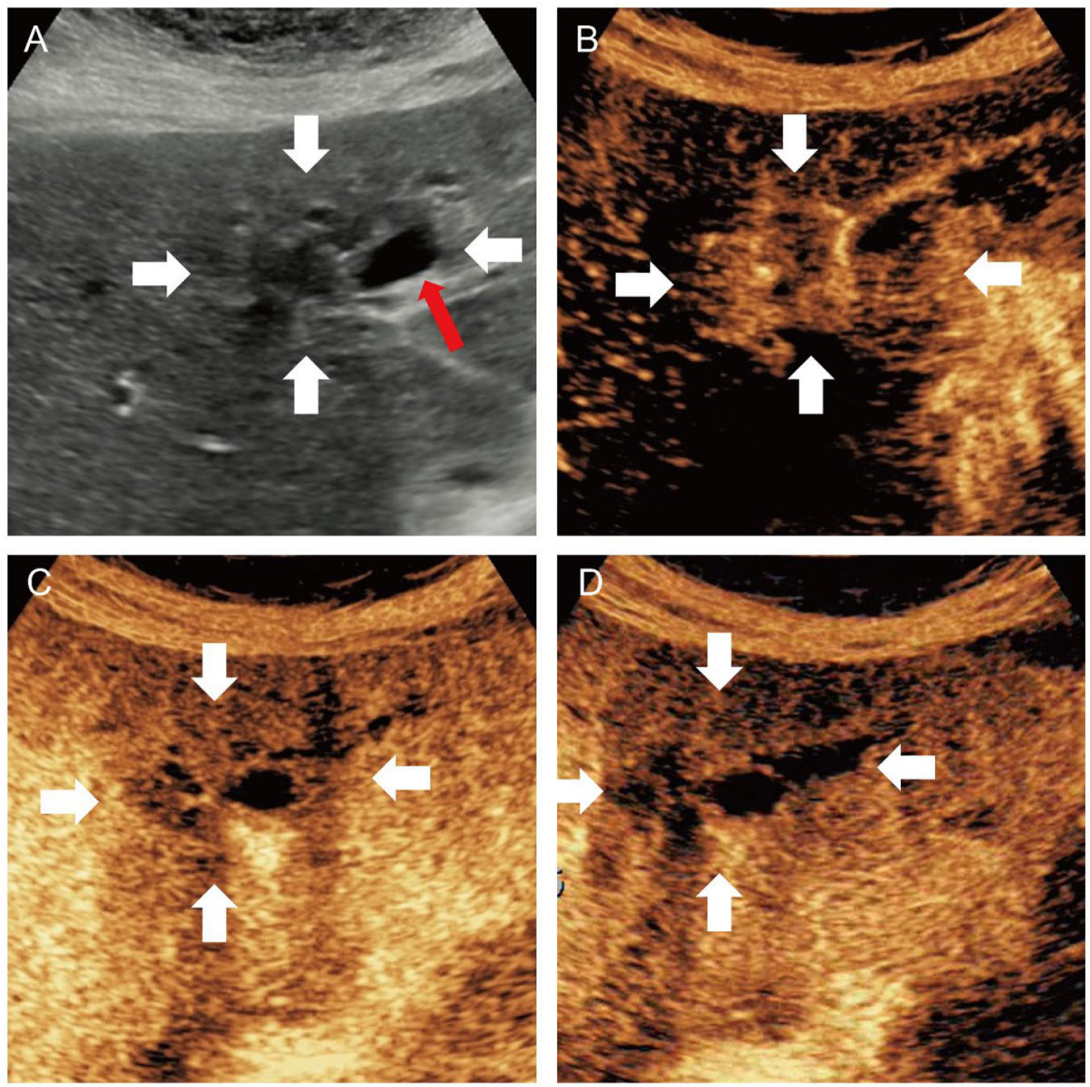
A 79-year-old female with a 14-mm biliary type MF-ICC. B-mode ultrasound showed hypo-echogenicity lesion and dilated bile duct (**A**). Circular enhancement was observed in the arterial phase (**B**). Hypo-enhancement was observed in the portal venous phase (**C**) and late phase (**D**). White arrow indicated the margin of the lesion; red arrow indicated the dilated bile duct

**Fig. 3:**
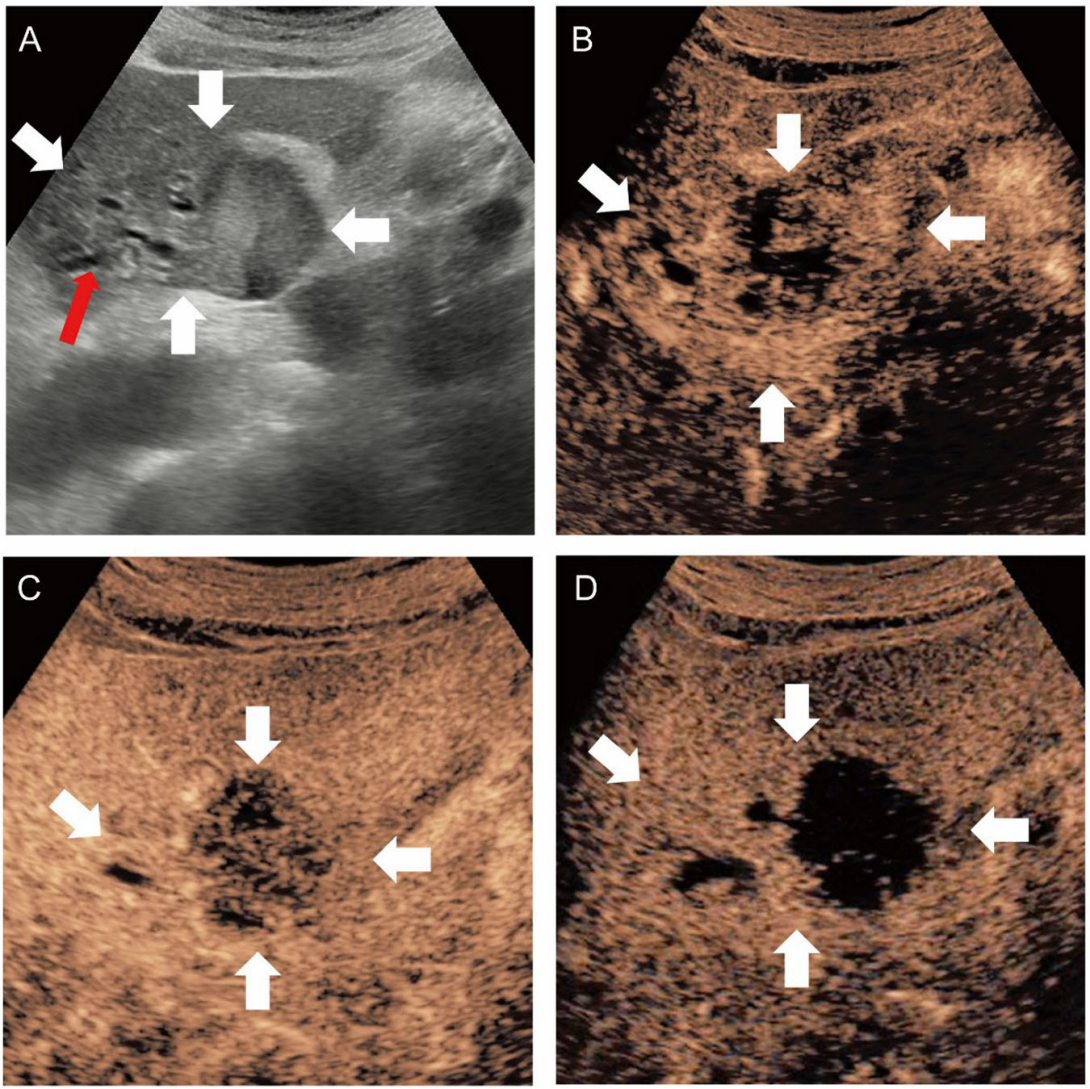
A 70-year-old male with a 32-mm biliary type MF-ICC. B-mode ultrasound showed hypo-echogenicity lesion and dilated bile duct (**A**). Circular enhancement was observed in the arterial phase (**B**). Hypo-enhancement was observed in the portal venous phase (**C**) and late phase (**D**). White arrow indicated the margin of the lesion; red arrow indicated the dilated bile duct

**Fig. 4:**
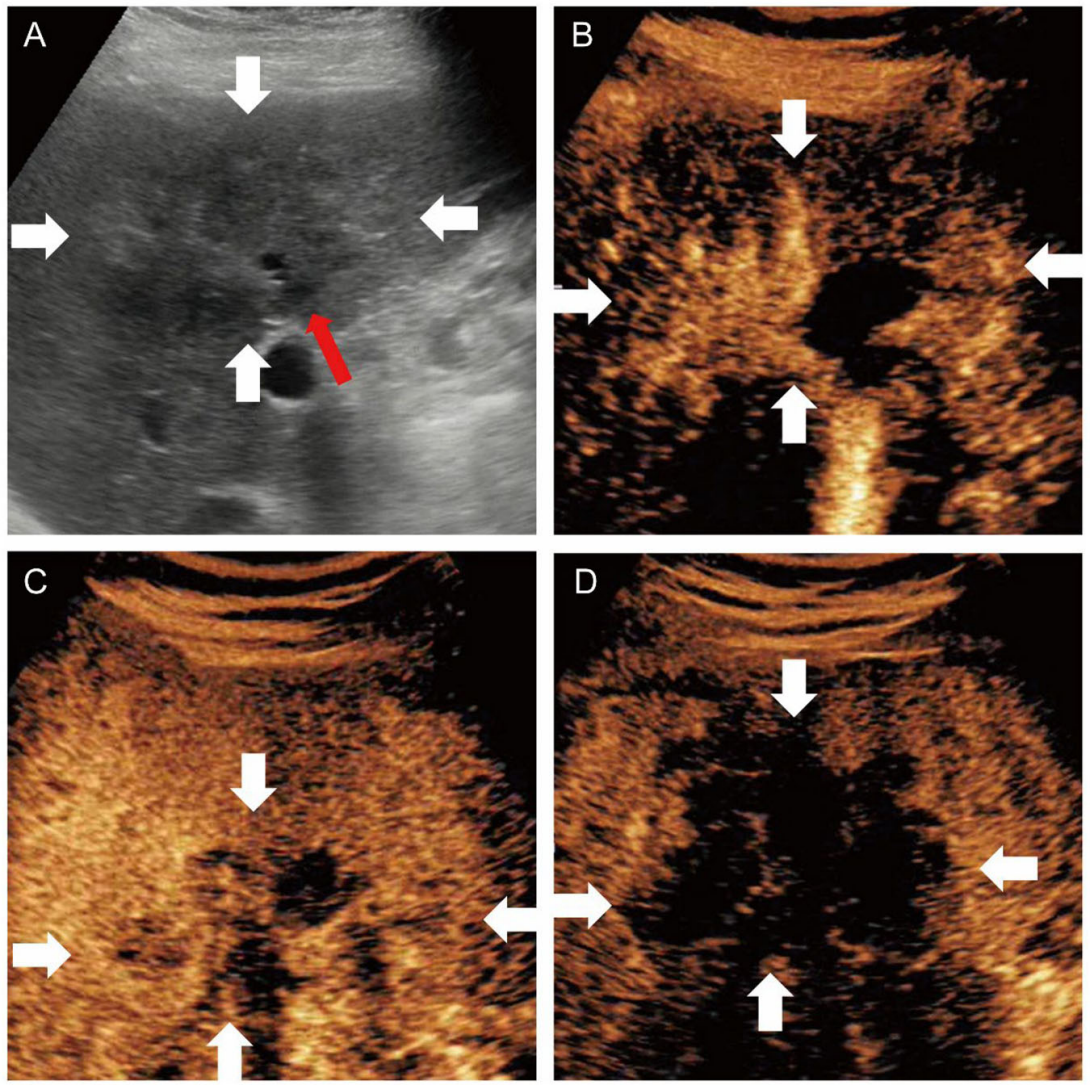
A 76-year-old female with a 56-mm biliary type MF-ICC. B-mode ultrasound showed hypo-echogenicity lesion and dilated bile duct (**A**). Non-circular enhancement was observed in the arterial phase (**B**). Hypo-enhancement was observed in the portal venous phase (**C**) and late phase (**D**). White arrow indicated the margin of the lesion; red arrow indicated the dilated bile duct

**Fig. 5:**
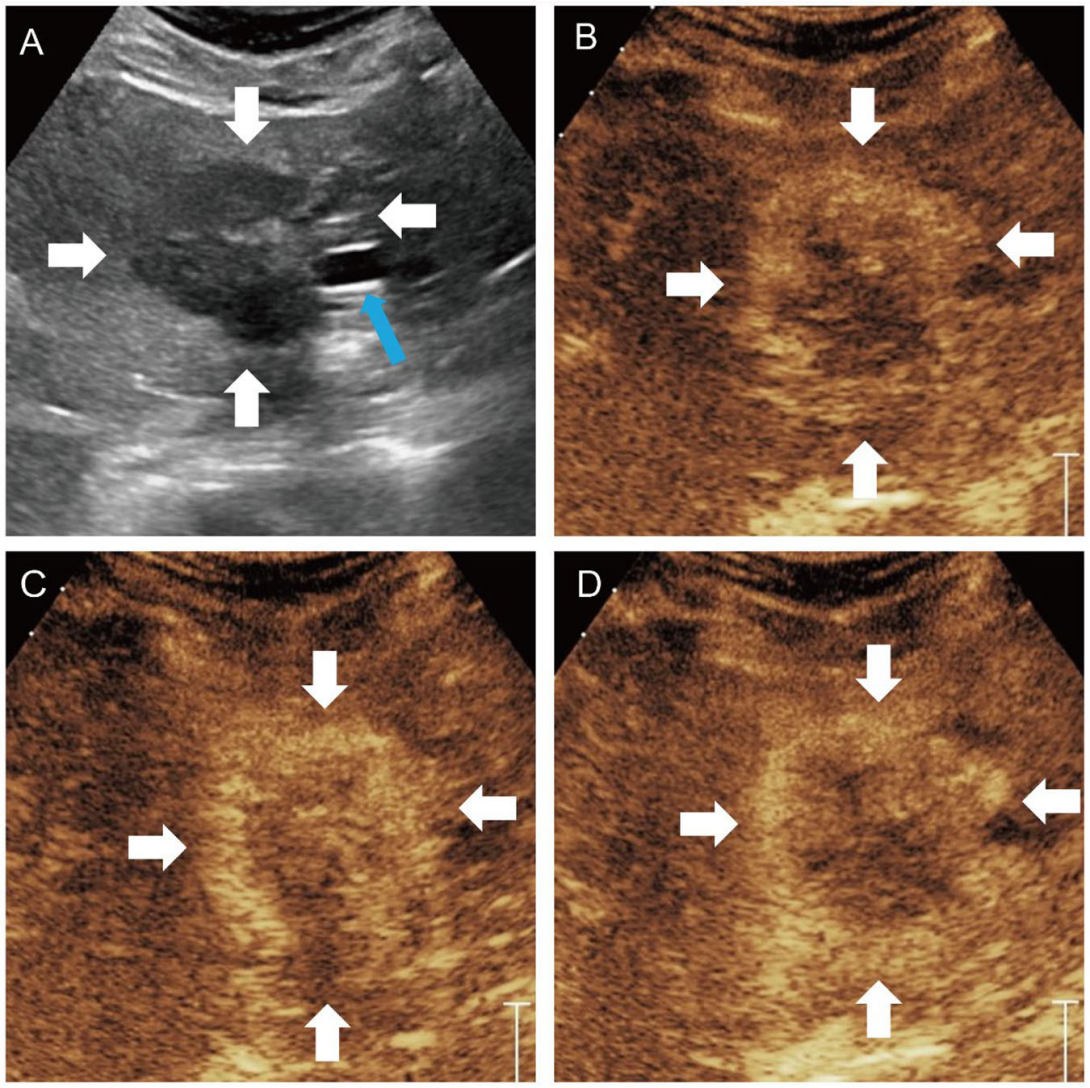
A 41-year-old female with 41-mm parenchymal type MF-ICC. B-mode ultrasound showed hypo-echogenicity lesion (**A**). Circular hyperenhancement was observed in the arterial phase (**B**). Hypo-enhancement was observed in the portal venous phase (**C**) and late phase (**D**). White arrow indicated the margin of the lesion; blue arrow indicated the left portal vein

Three sonographers subclassified MF-ICC as bMF-ICC (Figs. 2–4) or pMF-ICC (Fig. 5) based on preoperative US images. bMF-ICC was strictly defined by the presence of intra-tumoral bile duct dilatation, in which dilated bile ducts were entirely confined within the tumor mass. Otherwise, MF-ICCs were defined as pMF-ICC.

### Baseline characteristics collection

Clinicopathological data of the patients were collected from medical records, including age, sex, serum tumor markers (AFP, CEA, and CA19-9), hepatitis B virus (HBV), cirrhosis, pathological grade (G1, G2, and G3) [16], pathological classification (small and large duct types), Ki-67 expression, and postsurgical outcomes. Ki-67 nuclear staining with a positive rate ≥ 50% was defined as high expression, whereas a positive rate < 50% was defined as low expression [17].

### Statistical analysis

IBM SPSS Statistics (version 23.0, IBM Corporation, Armonk, USA) was used for the statistical analysis. We use the Fleiss Kappa coefficient to assess agreement between the readers. The level of agreement was considered as follows: 0.01–0.20 = poor agreement; 0.21–0.40 = fair agreement; 0.41–0.60 = moderate agreement; 0.61–0.80 = substantial agreement; 0.81–1.00 = excellent agreement [18]. Categorical variables were compared using the Chi-Squared test or Fisher’s exact test. Continuous variables were compared using *t*-test or Mann–Whitney U test. Survival rates were calculated using the Kaplan–Meier method and compared using the Breslow test. Statistical significance was set at *p* < 0.05.

## Results

### Baseline characteristics

The mean age of the patients was 61.96 ± 10.15 years, and 83 (58.9%) were men. The median size of the lesions was 47.00 mm (IQR 29.00–65.50 mm). Notably, 48 patients (34.5%) were HBV-positive, and 13 (9.3%) had cirrhosis. Perineural invasion was present in 46 lesions (33.8%), and lymph node metastasis was observed in 22 lesions (15.6%). Furthermore, 29 (20.6%) and 22 lesions (15.6%) had microvascular and bile duct invasions, respectively. Histologically, 37 lesions (27.4%) were classified as the large duct type. Additionally, 63 lesions (44.7%) exhibited a high Ki-67 expression. Detailed characteristics are shown in Table 1.

**Table 1:** Comparison of clinical features between bMF-ICC and pMF-ICC

Clinical features	Total	bMF-ICC	pMF-ICC	*p*-value
	*n* = 141	*n* = 33 (23.4%)	*n* = 108 (76.6%)	
Age (years)	61.96 ± 10.15	61.18 ± 10.31	62.20 ± 10.14	0.30
Sex, male/female (%) Tumor markers	83 (58.9%)/58 (41.1%)	22 (66.7%)/11 (33.3%)	61 (56.5%)/47 (43.5%)	0.29
AFP ≥ 20 µg/L^a^	11 (8.0%)	3 (9.4%)	8 (7.6%)	1.00
AFP < 20 µg/L	126 (92.0%)	29 (90.6%)	97 (92.4%)	
CEA ≥ 5 µg/L^a^	35 (25.5%)	14 (42.4%)	21 (20.2%)	0.01*
CEA < 5 µg/L	102 (74.5%)	19 (57.6%)	83 (79.8%)	
CA19-9 ≥ 34 µg/L^a^	48 (34.8%)	14 (42.4%)	34 (32.4%)	0.29
CA19-9 < 34 µg/L	90 (65.2%)	19 (57.6%)	71 (67.6%)	
HBsAg (+) (%)^a^	48 (34.5%)	11 (33.3%)	37 (34.9%)	0.87
Cirrhosis (%)^a^	13 (9.3%)	1 (3.0%)	12 (11.2%)	0.28
Tumor size (mm), median (IQR)	47.00 (29.00–65.50)	49.00 (32.00–81.50)	46.00 (29.00–61.75)	0.32
Leukocyte, median (IQR) (*10^9^)	6.28 (4.76–7.28)	6.45 (5.46–7.47)	6.06 (4.51–7.28)	0.29
Neutrophil, median (IQR) (*10^9^)	3.70 (2.70–4.66)	3.65 (3.10–4.69)	3.80 (2.60–4.60)	0.20
Pathological grading^a^				0.78
G1	0	0	0	
G2	107 (77.0%)	26 (78.8%)	81 (75.7%)	
G3	33 (23.6%)	7 (21.2%)	26 (24.3%)	
Pathological classification, Small/large duct type (%)^a^	98 (72.6%)/37 (27.4%)	7 (21.9%)/25 (78.1%)	91 (88.3%)/12 (11.7%)	< 0.001*
Microvascular invasion (%)	29 (20.6%)	18 (54.5%)	11 (10.2%)	< 0.001*
Multiple tumors (%)^a^	21 (15.4%)	8 (26.7%)	13 (12.3%)	0.10
Perineural invasion (%)^a^	46 (33.8%)	13 (44.8%)	33 (30.8%)	0.16
Lymph node metastasis (%)	22 (15.6%)	16 (48.5%)	6 (5.6%)	< 0.001*
Bile duct invasion (%)	22 (15.6%)	16 (48.5%)	6 (5.6%)	< 0.001*
Hepatic capsule invasion (%)^a^	50 (39.1%)	14 (53.8%)	36 (36.3%)	0.08
Ki-67 (+)^a^ (%)^b^	63 (44.7%)	21 (63.6%)	42 (38.9%)	0.01*

*IQR* interquartile range, *AFP* alpha-fetoprotein, *CEA* carcinoembryonic antigen, *CA19-9* carbohydrate antigen 19-9

* *p*-value < 0.05

^a^ Serum AFP, CEA, CA19-9, HBsAg (+), cirrhosis, pathological grading, pathological classification, multiple tumors, perineural invasion, hepatic capsule invasion and Ki-67 have missing values in 4, 4, 3, 2, 1, 1, 6, 5, 5, 13 and 1 cases, respectively

^b^ Ki-67 (+) indicates high Ki-67 expression

### Differences in clinicopathological features of bMF-ICC and pMF-ICC

Among 141 MF-ICC lesions that were reviewed by reviewer 1, 33 were categorized as bMF-ICCs. Whereas reviewer 2 categorized 42 lesions as bMF-ICCs. Additionally, reviewer 3 categorized 39 lesions as bMF-ICCs. The kappa coefficient was 0.808, indicating that the interobserver agreement for lesion subclassification was substantial. The reviewers’ opinions differed in 16 of the 141 cases (11.3%). Finally, after consensus, 33 lesions were categorized as biliary type (23.4%). Compared with pMF-ICCs, bMF-ICCs showed more frequent CEA ≥ 5 µg/L (*p* = 0.01), microvascular invasion (*p* < 0.001), lymph node metastasis (*p* < 0.001), bile duct invasion (*p* < 0.001) and high Ki-67 expression (*p* = 0.01). According to the pathological classification, bMF-ICCs tended to be of the large duct type (*p* < 0.001). Detailed characteristics are shown in Table 1*.*

ICC in stage T1 was subdivided into stages T1a and T1b depending on whether the tumor size was < 50 mm. Multivariate analysis showed that tumor size was an independent risk factor for postoperative mortality and recurrence in ICC, especially in patients with tumors > 50 mm [19, 20]. To further validate the differences between the biliary and parenchymal types, we performed a subgroup analysis based on tumor size (50 mm) (Table 2). After the subgroup analysis, we arrived at a similar conclusion. In tumors that were ≤ 50 mm, biliary type was associated with large duct type (*p* < 0.001), microvascular invasion (*p* < 0.001), lymph node metastasis (*p* < 0.001), and bile duct invasion (*p* < 0.001). Tumors > 50 mm in size were associated with the large duct type (*p* < 0.001), microvascular invasion (*p* = 0.001), multiple tumors (*p* = 0.007), perineural invasion (*p* = 0.006), lymph node metastasis (*p* = 0.009), bile duct invasion (*p* = 0.001), and high Ki-67 expression (*p* = 0.02).

**Table 2:** Comparison of clinicopathological features between bMF-ICC and pMF-ICC by subgroup analysis

	Tumor size ≤ 50 mm (*n* = 79)			Tumor size > 50 mm (*n* = 62)		
Clinical features	bMF-ICC (*n* = 18)	pMF-ICC (*n* = 61)	*p*-value	bMF-ICC (*n* = 15)	pMF-ICC (*n* = 47)	*p*-value
Age (years)	63.67 ± 10.41	62.46 ± 10.89	0.68	58.20 ± 9.69	61.87 ± 9.18	0.19
Sex (male/female, %)	13 (72.2%)/5 (27.8%)	43 (70.5%)/18 (29.5%)	0.89	9 (60.0%)/6 (40.0%)	18 (38.3%)/29 (61.7%)	0.14
Tumor markers						
AFP ≥ 20 µg/L^a^	1 (5.6%)	1 (1.7%)	0.41	2 (14.3%)	7 (15.6%)	1.00
AFP < 20 µg/L	17 (94.4%)	59 (98.3%)		12 (85.7%)	38 (84.4%)	
CEA ≥ 5 µg/L^b^	6 (33.3%)	11 (18.6%)	0.32	8 (53.3%)	10 (22.2%)	0.051
CEA < 5 µg/L	12 (66.7%)	48 (81.4%)		7 (46.7%)	35 (77.8%)	
CA19-9 ≥ 34 µg/L^a^	8 (44.4%)	18 (30.0%)	0.25	6 (40.0%)	16 (35.6%)	0.76
CA19-9 < 34 µg/L	10 (55.6%)	42 (70.0%)		9 (60.0%)	29 (64.4%)	
HBsAg (+) (%)^a^	4 (22.2%)	26 (43.3%)	0.11	7 (46.7%)	11 (23.9%)	0.18
Cirrhosis (%)^a^	0	10 (16.7%)	0.15	1 (6.7%)	2 (4.3%)	1.00
Leukocyte, median (IQR) (10^9^)	6.29 (4.95–7.66)	5.34 (4.49–7.25)	0.38	6.45 (5.71–7.05)	6.39 (4.87–7.59)	0.60
Neutrophil, median (IQR) (*10^9^)	3.30 (2.90–4.50)	3.20 (2.26–4.56)	0.39	3.96 (3.50–4.90)	4.00 (3.00–4.72)	0.63
Pathological classification, small/large duct type (%)^a^	1 (5.9%)/16 (94.1%)	49 (84.5%)/9 (15.5%)	< 0.001*	6 (40.0%)/9 (60.0%)	42 (93.3%)/3 (6.7%)	< 0.001*
Pathological grading^a^			0.78			1.00
G1	0	0		0	0	
G2	14 (77.8%)	44 (74.6%)		12 (80.0%)	37 (78.7%)	
G3	4 (22.2%)	15 (25.4%)		3 (20.0%)	10 (21.3%)	
Microvascular invasion (%)	10 (55.6%)	7 (11.5%)	< 0.001*	8 (53.3%)	4 (8.5%)	0.001*
Multiple tumors (%)^a^	1 (5.6%)	8 (13.6%)	0.77	7 (46.7%)	5 (10.6%)	0.007*
Perineural invasion (%)^a^	3 (21.4%)	20 (33.3%)	0.59	10 (66.7%)	13 (27.7%)	0.006*
Lymph node metastasis (%)	11 (61.1%)	4 (6.6%)	< 0.001*	5 (33.3%)	2 (4.3%)	0.009*
Bile duct invasion (%)	9 (50.0%)	3 (4.9%)	< 0.001*	7 (46.7%)	3 (6.4%)	0.001*
Hepatic capsule invasion (%)^a^	6 (46.2%)	19 (34.5%)	0.65	8 (61.5%)	17 (36.2%)	0.10
Ki-67 (+)^a^ (%)^b^	10 (55.6%)	24 (39.3%)	0.22	11 (73.3%)	18 (38.3%)	0.02*

*IQR* interquartile range, *AFP* alpha-fetoprotein, *CEA* carcinoembryonic antigen, *CA19-9* carbohydrate antigen 19-9

* *p*-value < 0.05

^a^ Serum AFP, CEA, CA19-9, HBsAg (+), cirrhosis, pathological grading, pathological classification, multiple tumors, perineural invasion, hepatic capsule invasion and Ki-67 have missing values in 4, 4, 3, 2, 1, 1, 6, 5, 5, 13 and 1 cases, respectively

^b^ Ki-67 (+) indicates high Ki-67 expression

### Differences in ultrasound features of bMF-ICC and pMF-ICC

Considering tumor location, bMF-ICCs were more frequently located in the left lobe of the liver (*p* = 0.03) than in the parenchymal type. There were no statistically significant differences in other US features between bMF-ICCs and pMF-ICCs. Detailed characteristics are listed in Table 3*.*

**Table 3:** Comparison of ultrasound characteristics between bMF-ICC and pMF-ICC

Ultrasound characteristics	Total (*n* = 141)	bMF-ICC (*n* = 33)	pMF-ICC (*n* = 108)	Statistic	*p*-value
Tumor location (%)				χ^2^ = 4.90	0.03*
Left lobe	65 (46.1%)	21 (63.6%)	45 (41.7%)		
Right lobe	76 (53.9%)	12 (36.4%)	63 (58.3%)		
Grayscale ultrasound					
Echogenicity (%)				χ^2^ = 3.67	0.16
Hyper-echogenicity	14 (9.9%)	4 (12.1%)	10 (9.3%)		
Iso-echogenicity	11 (7.8%)	5 (15.2%)	6 (5.6%)		
Hypo-echogenicity	116 (82.3%)	24 (72.7%)	92 (85.2%)		
Shape (%)				χ^2^ = 1.22	0.27
Regular	36 (25.5%)	6 (18.2%)	30 (27.8%)		
Irregular	105 (74.5%)	27 (81.8%)	78 (72.2%)		
Margin (%)				χ^2^ = 1.08	0.30
Clear	58 (41.1%)	11 (33.3%)	47 (43.5%)		
Ill-defined	83 (58.9%)	22 (66.7%)	61 (56.5%)		
CEUS					
The initial enhancement time (s) (IQR)	17.00 (13.00–21.00)	20.00 (14.50–21.00)	17.00 (13.00–20.75)	z = −1.727	0.08
Time to peak (s) (IQR)	25.00 (20.00–30.00)	27.00 (22.00–32.00)	24.00 (20.00–28.00)	z = −1.715	0.09
The washout time (s) (IQR)	39.00 (32.00–50.00)	42.00 (31.50–54.50)	38.00 (32.00–49.50)	z = −1.267	0.21
Contrast enhancement pattern (%)				χ^2^ = 0.30	0.86
Circular enhancement	67 (47.5%)	17 (51.5%)	50 (46.3%)		
Non-circular enhancement	52 (36.9%)	11 (33.3%)	41 (38.0%)		
Nodular enhancement	22 (15.6%)	5 (15.2%)	17 (15.7%)		
Arterial phase enhancement degree (%)				χ^2^ = 0.32	0.85
Hyper-enhancement	132 (93.6%)	31 (93.9%)	101 (93.5%)		
Iso-enhancement	6 (4.3%)	1 (3.0%)	5 (4.6%)		
Hypo-enhancement	3 (2.1%)	1 (3.0%)	2 (1.9%)		
Portal venous phase enhancement degree (%)				χ^2^ = 0.34	0.86
Hyper-enhancement	0	0	0		
Iso-enhancement	5 (3.5%)	1 (3.0%)	4 (3.7%)		
Hypo-enhancement	136 (96.5%)	32 (97.0%)	104 (96.3%)		
Late-phase enhancement degree (%)				χ^2^ = 0.80	0.37
Hyper-enhancement	0	0	0		
Iso-enhancement	2 (1.4%)	1 (3.0%)	4 (3.7%)		
Hypo-enhancement	139 (98.6%)	32 (97.0%)	104 (96.3%)		
Degree of clearance				χ^2^ = 0.18	0.67
No clearance	0	0	0		
Slight clearance	130 (93.6%)	31 (93.9%)	99 (91.7%)		
Significant clearance	11 (7.8%)	2 (6.1%)	9 (8.3%)		

* *p*-value < 0.05

### Differences in postsurgical outcomes of bMF-ICC and pMF-ICC

**Fig. 6:**
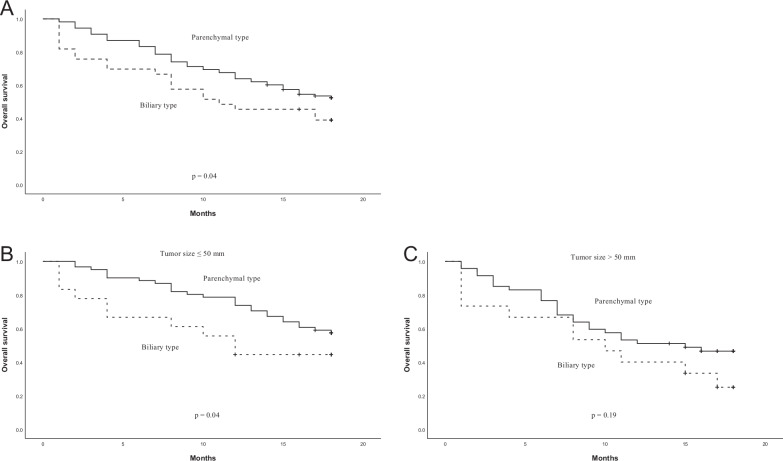
Postsurgical outcomes of bMF-ICC and pMF-ICC. Kaplan–Meier plot of overall survival (**A**). Subgroup analysis for overall survival by tumor size ≤ 50 mm (**B**), and > 50 mm (**C**)

We compared the 18-month overall survival between pMF-ICC and bMF-ICC and found that pMF-ICC showed better overall survival (*p* = 0.04) (Fig. 6A). Our subgroup analysis revealed that in MF-ICC ≤ 50 mm, pMF-ICCs had better overall survival (*p* = 0.04) (Fig. 6B). However, for MF-ICCs > 50 mm, no statistically significant difference in overall survival was observed (*p* = 0.19) (Fig. 6C).

## Discussion

In this study, we categorized MF-ICCs into bMF-ICCs and pMF-ICCs based on US imaging and compared the clinicopathological features and postoperative outcomes of the two types. Our study showed that bMF-ICC was associated with CEA ≥ 5 µg/L, microvascular invasion, lymph node metastasis, bile duct invasion, and high Ki-67 expression (all *p* < 0.05). pMF-ICCs showed better overall survival at follow-up (*p* = 0.04). The subgroup analysis showed that in MF-ICC cases ≤ 50 mm, pMF-ICCs showed better survival (*p* = 0.04). Anatomically, ICCs derived from the second or segmentalized branch of the large bile duct are usually considered to be of the large duct type [5, 21]. Aishima et al classified ICCs < 50 mm into hilar and peripheral types according to their anatomical location [22]. However, for ICCs > 50 mm in size, classification based on anatomical location is not suitable. Therefore, a classification based on bile duct abnormalities is more suitable for clinical management.

Furthermore, our study showed that bMF-ICCs tended to be of the large duct type in pathological classification. bMF-ICCs often exhibited aggressive pathological features, which were like the MRI classification by Rhee et al, demonstrating that some characteristics that had been described in MRI classification were transposable to ultrasound classification [12]. The American Joint Committee on Cancer Staging System is an important tool for assessing the prognosis of patients with cancer [23]. Our subgroup analysis showed that bMF-ICCs and pMF-ICCs had different prognoses for T1a stage MF-ICCs. Moreover, in MF-ICC cases ≤ 50 mm and > 50 mm, bMF-ICCs were pathologically more likely to be classified as a large duct type. This subclassification method may serve as a non-invasive method for predicting the pathological classification of MF-ICCs. The majority of patients with ICC are deemed unresectable at initial diagnosis due to factors such as multifocal tumors and lymph node metastasis [24]. In our study, we observed that among MF-ICC ≤ 50 mm, bMF-ICC is significantly associated with large duct type, microvascular invasion, lymph node metastasis, and bile duct invasion. In MF-ICC > 50 mm, bMF-ICC demonstrates even more aggressive biological behavior, exhibiting not only the aforementioned features but also a higher incidence of multifocality, perineural invasion, and high Ki-67 expression. These findings suggest that prompt surgical intervention is crucial to prevent progression to an unresectable state for bMF-ICC. Furthermore, given the higher propensity for lymphatic spread, a more extensive lymphadenectomy is warranted during surgery. Lymphadenectomy confers notable prognostic benefit in patients with less advanced ICC and plays an essential role in accurate pathological staging [25]. In contrast, patients with pMF-ICC are more likely to achieve favorable survival outcomes following surgical resection. We believe that this may be due to high Ki-67 expression in bMF-ICCs. Previous research has shown that Ki-67 is a nuclear antigen associated with cell proliferation [26]. The high proliferative ability of tumor cells may promote the formation of new blood vessels, thereby enhancing tumor invasiveness. High Ki-67 levels have been correlated with poor survival in cholangiocarcinoma [27].

Additionally, bMF-ICCs were mostly located in the left lobe; we speculate that the reason is that the left hepatic duct is longer and runs more vertically, and the drainage angle of the left hepatic duct is sharper [28]. These features lead to the mechanical compression of the duct lumen, resulting in secondary bile duct dilation proximal to the stenotic segment. Another reason is that the left lobe is more prone to bile duct stone formation, which can lead to mechanical bile duct dilation [29]. CEUS plays an important role in the diagnosis of ICC. ICC often presents with early enhancement and washout, while the tumor margin shows more prominent enhancement than the center, typically exhibiting a circular enhancement pattern [30]. Our study findings are consistent with this, with approximately 47.5% of the cases showing circular enhancement. We aimed to study the changes in blood flow between the two types of MF-ICCs using CEUS; however, the results showed no significant differences between them.

High CEA levels indicate stronger tumor invasiveness, suggesting the presence of distant metastasis or microvascular invasion, both of which are indicative of a worse survival rate [17]. This is consistent with our findings. Therefore, our study provides a preoperative, non-invasive, and convenient way to obtain clinicopathological information related to prognosis that is easily distinguishable.

This study had some limitations. First, the sample size was relatively small because the incidence of ICC is relatively low. Therefore, further studies with larger sample sizes are required to effectively validate the clinicopathological features and postsurgical outcomes of the different subtypes. Second, owing to the availability of patient data, we collected consecutive patients from January 2020 to May 2023. Only short-term follow-up data were analyzed in our study. However, because of the subtle symptoms of ICC, most patients develop unresectable tumors or distant metastases at diagnosis, leading to an overall survival of only about 1 year [31]. Therefore, for most patients with ICC, we believe that short-term follow-up may have greater clinical significance than long-term monitoring. Further long-term studies are needed to analyze the long-term outcomes of the different subtypes. Third, the clinicopathological features and postsurgical outcomes of the different subtypes were not compared with those of the MRI-based subtype because of the retrospective nature of the dataset. Accordingly, a prospective study should be conducted to further investigate subtypes based on different imaging modalities. Fourth, this study was conducted in China, where ICC exhibits a distinct association with HBV infection [32], differing from other populations with low HBV infection rates. Therefore, our findings may not be generalizable to other populations. Future multicenter studies involving geographically diverse cohorts are warranted to validate the universality of our conclusions.

In conclusion, we propose a classification method for MF-ICCs based on ultrasound imaging, categorizing MF-ICCs into bMF-ICC and pMF-ICC. This approach is cost-effective and feasible, demonstrating substantial interobserver consistency. The two subtypes exhibit significant differences in clinicopathological features and postoperative outcomes. These findings provide important clinical value for future management strategies of MF-ICCs.

## Data Availability

The datasets used or analyzed during the current study are available from the corresponding author upon reasonable request.

## Abbreviations

bMF-ICC: Biliary type mass-forming intrahepatic cholangiocarcinoma
CA19-9: Carbohydrate antigen 19-9
CEA: Carcinoembryonic antigen
CEUS: Contrast-enhanced ultrasound
HBsAg: Surface antigen of the hepatitis B virus
HBV: Hepatitis B virus
ICC: Intrahepatic cholangiocarcinoma
IQR: Interquartile range
MF-ICC: Mass-forming intrahepatic cholangiocarcinoma
MRI: Magnetic resonance imaging
pMF-ICC: Parenchymal type mass-forming intrahepatic cholangiocarcinoma
US: Ultrasound

## Declarations

### Study design

This is a retrospective study.
